# Genomic measures of inbreeding in the Norwegian–Swedish Coldblooded Trotter and their associations with known QTL for reproduction and health traits

**DOI:** 10.1186/s12711-019-0465-7

**Published:** 2019-05-27

**Authors:** Brandon D. Velie, Marina Solé, Kim Jäderkvist Fegraeus, Maria K. Rosengren, Knut H. Røed, Carl-Fredrik Ihler, Eric Strand, Gabriella Lindgren

**Affiliations:** 1 School of Life and Environmental Sciences, University of Sydney, Sydney, Australia; 2 Department of Animal Breeding and Genetics, Swedish University of Agricultural Sciences, Uppsala, Sweden; 3 Department of Basic Sciences and Aquatic Medicine, Norwegian University of Life Sciences, Oslo, Norway; 4 Department of Companion Animal Clinical Sciences, Norwegian University of Life Sciences, Oslo, Norway; 5 Livestock Genetics, Department of Biosystems, KU Leuven, Leuven, Belgium

## Abstract

**Background:**

Since the 1950s, the Norwegian–Swedish Coldblooded trotter (NSCT) has been intensively selected for harness racing performance. As a result, the racing performance of the NSCT has improved remarkably; however, this improved racing performance has also been accompanied by a gradual increase in inbreeding level. Inbreeding in NSCT has historically been monitored by using traditional methods that are based on pedigree analysis, but with recent advancements in genomics, the NSCT industry has shown interest in adopting molecular approaches for the selection and maintenance of this breed. Consequently, the aims of the current study were to estimate genomic-based inbreeding coefficients, i.e. the proportion of runs of homozygosity (ROH), for a sample of NSCT individuals using high-density genotyping array data, and subsequently to compare the resulting rate of genomic-based F (F_ROH_) to that of pedigree-based F (F_PED_) coefficients within the breed.

**Results:**

A total of 566 raced NSCT were available for analyses. Average F_ROH_ ranged from 1.78 to 13.95%. Correlations between F_ROH_ and F_PED_ were significant (*P* < 0.001) and ranged from 0.27 to 0.56, with F_PED_ and F_ROH_ from 2000 to 2009 increasing by 1.48 and 3.15%, respectively. Comparisons of ROH between individuals yielded 1403 regions that were present in at least 95% of the sampled horses. The average percentage of a single chromosome covered in ROH ranged from 9.84 to 18.82% with chromosome 31 and 18 showing, respectively, the largest and smallest amount of homozygosity.

**Conclusions:**

Genomic inbreeding coefficients were higher than pedigree inbreeding coefficients with both methods showing a gradual increase in inbreeding level in the NSCT breed between 2000 and 2009. Opportunities exist for the NSCT industry to develop programs that provide breeders with easily interpretable feedback on regions of the genome that are suboptimal from the perspective of genetic merit or that are sensitive to inbreeding within the population. The use of molecular data to identify genomic regions that may contribute to inbreeding depression in the NSCT will likely prove to be a valuable tool for the preservation of its genetic diversity in the long term.

**Electronic supplementary material:**

The online version of this article (10.1186/s12711-019-0465-7) contains supplementary material, which is available to authorized users.

## Background

In recent years, there has been a rapid increase in the intensity of selection in many livestock breeding programs with the growing use of elite animals, which ultimately reduces the effective population size (N_e_) of some breeds [1–6]. Consequently, a small N_e_ not only reduces genetic variability, but it also increases the effects of inbreeding (F) and genetic drift, and potentially alters the patterns of runs of homozygosity (ROH) in the long term [3–8]. While such alterations may not necessarily be of concern for large and highly diverse populations, increased homozygosity at loci with a heterozygous advantage in small native populations reduces furthermore their genetic diversity [7]. Small populations can be particularly vulnerable to inbreeding depression since mating between relatives often decreases individual fitness and can significantly reduce population growth [7, 9]. Moreover, selection programs, while driving favorable alleles to fixation, also allow deleterious alleles to hitchhike along with favorable mutations. In addition to this, more intense selection resulting from combining genomic selection with embryo biotechnologies (e.g. artificial insemination) not only increases rates of genetic gain, but can also increase levels of inbreeding [4, 10].

The Norwegian–Swedish Coldblooded trotter (NSCT) is a domestic breed of horse in Norway and Sweden and is one of the few remaining descendants of the original Nordic coldblooded horse [11]. Since the 1950s, the breed has been intensively selected for harness racing performance with estimated breeding values produced annually since the 1980s [12, 13]. As a result, a remarkable improvement in the racing performance of NSCT has occurred during the last half-century. However, this improved racing performance has also been accompanied by a gradual increase in pedigree-based F levels [14]. Although today NSCT is considered as a relatively healthy breed, the NSCT breeding industry is well aware that increased levels of inbreeding are widely known to increase the expression of recessive deleterious alleles that are linked to genetic diseases. Historically, inbreeding in NSCT has been monitored by using traditional methods that are based on pedigree analysis [14, 15]. While informative, the NSCT industry understands that this classical metric likely underestimates inbreeding within the breed and does not account for the fact that homozygosity at some regions may, in principal, be more or less desirable than at other regions. Two animals that have the same level of inbreeding, may display drastically different unfavorable effects of inbreeding. Even with an extensive and complete pedigree, realized inbreeding levels will likely differ from pedigree-based F levels due to recombination and Mendelian sampling, which is then compounded by the fact that, although the base animals in a pedigree are considered unrelated, they are more often than not, related.

Consequently, the NSCT industry has actively supported a shift towards using genomic data for F calculations in the breed, thus allowing for diversity across the entire genome as well as at specific regions to be evaluated and monitored, and providing not only a more accurate assessment of inbreeding within the breed, but also a much more detailed assessment. As such, the aims of the current study were to provide genomic-based F coefficient estimates (F_ROH_) for a sample of NSCT using a high-density genotyping array and to compare the rate of F_ROH_ to that of classical pedigree-based F (F_PED_) within the breed. Common ROH within the breed were also assessed for overlaps with previously characterized quantitative trait loci (QTL) for health and reproduction traits in the horse, thus providing a first look at genomic regions and traits that may warrant industry intervention in the future.

## Methods

### Pedigree data

Complete pedigree information on all raced and unraced NSCT were provided by the trotter associations in both Norway and Sweden (Det Norske Travselskap and Svensk Travsport). The pedigree consisted of 112,195 individuals with a median pedigree depth of 15 generations.

### Collection of samples

In total, 566 individuals born between 1 January 2000 and 31 December 2009 were selected for this study based on the following criteria: (1) each horse had to have participated in at least one race during its lifetime; this restriction was implemented to allow for a broader use of the data in future analyses that will explore racing performance traits within the breed; (2) hair and/or blood samples had to be readily accessible from the pedigree registration authorities in either Norway (Department of Basic Sciences and Aquatic Medicine, Norwegian University of Life Sciences) or Sweden (Animal Genetics Laboratory, Swedish University of Agricultural Sciences); and (3) a sufficient amount of sample material had to be available to ensure high DNA quality standards.

### DNA isolation

DNA was extracted from hair roots using a standard procedure of hair preparation. Briefly, 186 μL of Chelex 100 Resin (Bio-Rad Laboratories, Hercules, CA) and 14 μL of proteinase K (20 mg/mL; Merck KgaA, Darmstadt, Germany) were added to each sample. This mix was incubated at 56 °C for 2 h and proteinase K was inactivated for 10 min at 95 °C. For DNA preparation from blood, DNA from 350-μL blood samples were extracted by using the Qiasymphony instrument and the Qiasymphony DSP DNA mini kit (Qiagen, Hilden, Germany).

### Genotyping and quality control

Prior to quality control (QC), the dataset consisted of individuals that were genotyped with the 670K Axiom equine genotyping array (n = 473) and the 670K+ Axiom equine genotyping array (n = 93). Data from the two arrays were subsequently merged based on SNP name, chromosome number and position, which yielded a combined SNP dataset of 611,888 SNPs for 566 horses (SNPs located on chromosomes X and Y were excluded during this process). Then, QC was performed with the PLINK v1.07 software. SNPs were screened based on minor allele frequency (MAF > 0.01), Hardy–Weinberg equilibrium (*p* > 0.0001), and genotyping rate (> 0.95) with data that did not conform to these criteria and individuals with missing genotypes (> 15%) being removed. Descriptive data for the sample of horses used in the analyses are in Table 1.

**Table 1:** Descriptive data on the genotyped horses

	Number
Sex	
Intact males	56
Females	222
Geldings	288
Country of birth	
Norway	265
Sweden	301
Year of birth	
2000	25
2001	60
2002	72
2003	53
2004	55
2005	40
2006	60
2007	70
2008	63
2009	68
Total	566

### Inbreeding coefficient and runs of homozygosity

Inbreeding coefficients (F_PED_) were calculated based on the complete pedigree of the breed using the Contribution, Inbreeding (F), Coancestry v1.0 software, which uses a modified algorithm of Sargolzaei et al. [16] to compute inbreeding coefficients that is a fast and accurate tool for F_PED_ calculations.

Inconsistency between ROH-defining criteria in various industries and breeds has been shown to convolute the comparison of studies over time and across population samples [1–6, 17–22]. Since the criteria to define a ROH continue to remain ambiguous, in our study, we applied a wide range of ROH-defining criteria. Runs of homozygosity were defined in PLINK v1.07 using the sliding windows approach through the *homozyg* command. The details of each applied threshold setting are in Table S1 (see Additional file 1: Table S1). Genomic inbreeding coefficients (F_ROH_) were estimated for each threshold setting by dividing the summed length of all ROH (per individual) by the length of the genome (2,242,879,462 bp) covered with SNPs. Pearson correlation coefficients between F_PED_ and all F_ROH_ were determined using the statistical software R [23]. Paired t-tests between all F_ROH_ were also performed.

To better identify population-wide ROH in the breed, custom scripts in R were applied to ROH data from the threshold setting that resulted in the highest correlation between F_PED_ and F_ROH_. These scripts were used to determine which regions of the genome were shared in at least 95% of individuals in the sample [23]. Ultimately, we chose the threshold setting that resulted in the highest correlation between F_PED_ and F_ROH_ since not only did it allow the capture of longer ROH that would subsequently be more beneficial when evaluating previously associated QTL, but it also yielded a more conservative estimate of inbreeding within the breed (i.e. an estimate that was more likely to be skewed upwards than downwards). Homozygous regions that were present in at least 95% of the sampled NSCT were then compared to previously reported QTL for reproduction and health traits in the horse (downloaded from the horse QTL database; [24]) using bed file comparisons in BEDOPS [25].

## Results

**Fig. 1:**
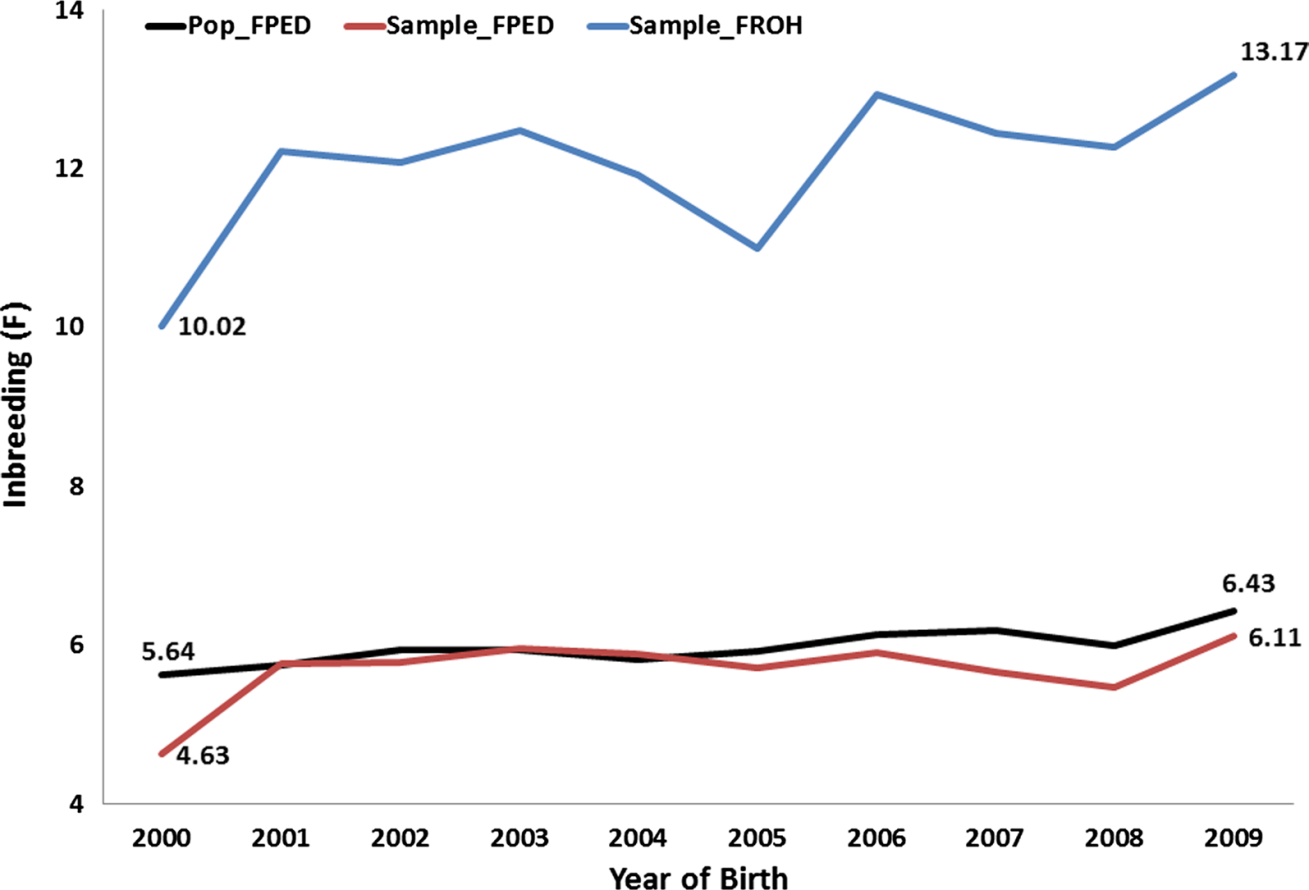
Median inbreeding levels in the Norwegian–Swedish Coldblooded trotter for horses born between 2000 and 2009. Pop_FPED = pedigree inbreeding for the population (n = 14,547); Sample_FPED = pedigree inbreeding and Sample_FROH = genomic inbreeding for the sample of individuals studied here (n = 566)

**Fig. 2:**
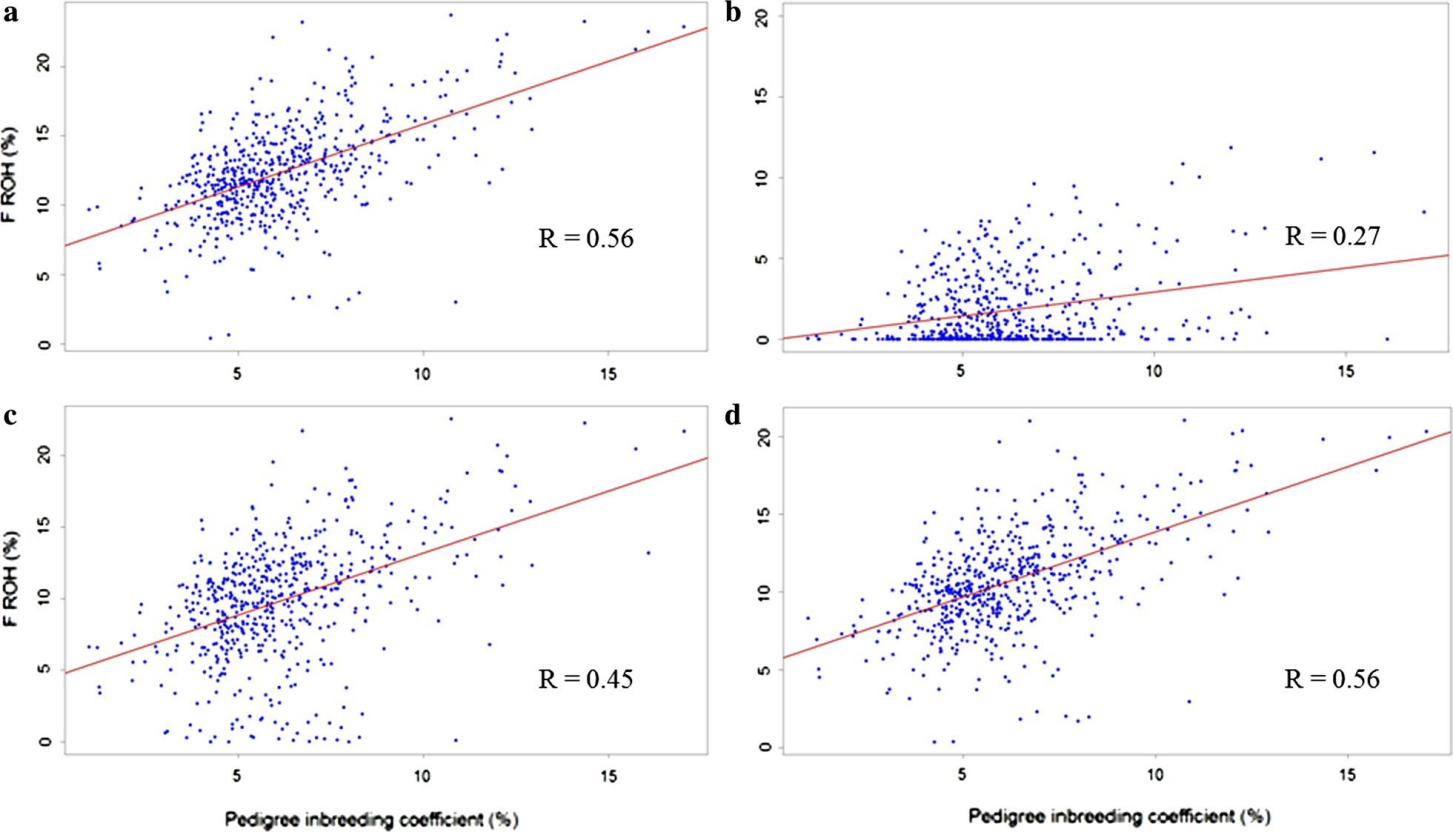
Scatterplot of genomic inbreeding coefficient (F_ROH_) on pedigree inbreeding coefficient (F_PED_). A = 50snp_500 kb _100snp_1_5; B = 500snp_500 kb _50snp_0_0; C = 50snp_500 kb _100snp_0_1; D = pruned_50snp_500 kb _50snp_1_2 (FORMAT: “sliding window size”_”minimum length (kb) for a run to be called as a ROH”_”minimum number of SNPs for a run to be called as a ROH”_”number of heterozygous SNPs allowed in a ROH”_”number of missing calls allowed in a ROH”)

After QC, 360,977 autosomal SNPs and 566 horses were available for analyses. Summary statistics, stratified by country of birth, for F_PED_ are in Table 2. F_PED_ and F_ROH_ of Norwegian born horses were higher than those of Swedish born horses, although the highest F_PED_ estimate was found for a Swedish born horse. Median F_PED_ and F_ROH_ for the entire cohort of sampled horses, stratified by year, are shown in Fig. 1. Inbreeding in the NSCT population during the 2000–2009 period increased by 1.48 and 3.15% based on F_PED_ and F_ROH_ estimates, respectively. Average F_ROH_ (%) ranged from 1.78 to 13.95% (see Additional file 1: Table S1). Correlations between F_PED_ and all F_ROH_ estimates were significant (*P* < 0.001) and ranged from 0.27 to 0.56 (see Additional file 2: Table S2) and Fig. 2. The threshold settings as defined below resulted in the highest correlation (R = 0.5629) between F_PED_ and F_ROH_:

- Size of the sliding window in SNPs: 50 SNPs.
- Minimum length in kb that a run must have to be called as a ROH: 500.
- Minimum number of SNPs that a run must have to be called as a ROH: 100.
- Number of heterozygous SNPs allowed in a ROH: 1.
- Number of missing calls allowed in a ROH: 5.
- Pruned for linkage disequilibrium: No.
- Minimum density to consider a ROH: 1 SNP per 50 kb.
- Maximum gap allowed between two SNPs: 100 kb.

**Table 2:** Descriptive results, stratified by country of birth, for average inbreeding coefficient (F_PED_) and average genomic inbreeding coefficient (F_ROH_) for a sample of raced Norwegian–Swedish Coldblooded trotters born between 1 January 2000 and 31 December 2009

	Min	25th percentile	Median	Mean	75th percentile	Max
Country of birth Norway						
F_PED_ (%)	0.96	5.18	6.18	6.59	7.38	14.35
F_ROH_ (%)^a^	1.98	8.86	10.12	9.60	11.76	14.39
Country of birth Sweden						
F_PED_ (%)	1.19	4.50	5.42	5.81	6.86	17.04
F_ROH_ (%)^a^	1.61	7.39	9.03	8.69	10.98	13.56

^a^Results based on the F_ROH_ across all threshold settings

Whereas the above settings resulted in the highest correlation between F_PED_ and F_ROH_, a similarly strong correlation (R = 0.5594) was obtained from the analysis of the pruned data with the same threshold settings except that the minimum number SNPs that a run must have to be called as a ROH was set to 50 SNPs.

Paired *t* test between all F_ROH_ yielded significant differences for most of the F_ROH_ threshold settings with only 35 (1.49%) comparisons resulting in no significant difference (see Additional file 3: Table S3). Variations in sliding window size, minimum length in kb and minimum number of SNPs of a run to be called as a ROH clearly altered F_ROH_. The influence of different threshold settings on ROH length and ultimately on F_ROH_ is illustrated in Figures S1 and S2 (see Additional file 4: Figure S1 and file 5 Figure S2).

**Fig. 3:**
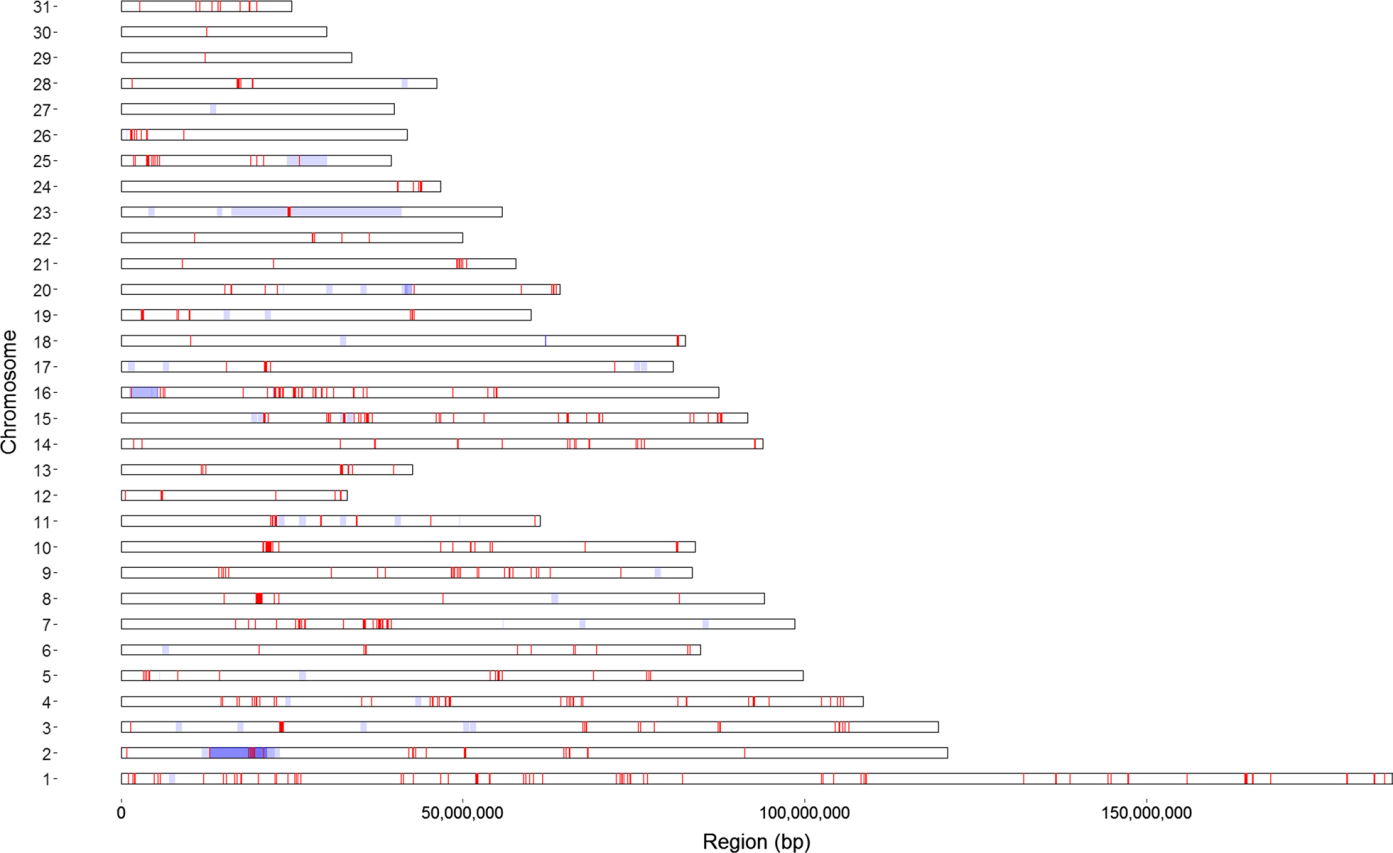
Location of runs of homozygosity (ROH) across the horse chromosomes tha are common to 95% of the sampled Norwegian–Swedish Coldblooded Trotter population. Regions containing previously characterized QTL for reproduction and health traits are shaded in blue with the shade of blue reflecting the number of QTL in the region (darker as the number of QTL increases)

By applying the threshold settings that resulted in the highest correlation between F_PED_ and F_ROH_, the average percentage of a single chromosome covered in ROH ranged from 9.84 to 18.82% (Table 3 Column D). Comparisons of ROH between individuals yielded 1403 regions that were present in at least 95% of the sampled horses (Fig. 3). The length of these regions ranged from 1 bp to 935 kb and overlapped with 35 previously characterized QTL for reproduction and health traits (see Additional file 6: Table S4). A visual representation of overlapping regions is in Fig. 3 with a brief description of each overlapped QTL in Table 4. QTL associated with osteochondrosis accounted for 48.6% of the overlapped QTL with only one of the 35 QTL being associated with fertility (QTL 103450, located on *Equus caballus* chromosome (ECA) 1).

**Table 3:** Average percentage of the genome, stratified by chromosome, covered by runs of homozygosity (ROH)

Chromosome	Average ROH (%)							
	A	B	C	D	E	F	G	H
1	10.08	12.67	14.89	15.05	13.59	13.58	7.35	11.34
2	9.40	11.67	13.89	14.08	12.02	12.02	8.62	10.74
3	9.18	11.07	12.66	12.74	12.27	12.24	10.02	9.11
4	10.09	12.27	13.82	13.99	13.16	13.17	11.50	10.61
5	10.38	12.28	14.28	14.46	13.06	13.05	10.62	11.10
6	8.30	10.34	12.58	12.69	10.57	10.57	8.21	10.23
7	10.41	12.23	14.29	14.48	13.12	13.07	9.53	9.58
8	8.61	10.07	11.02	11.05	10.43	10.45	11.73	8.93
9	9.25	10.88	12.90	13.05	11.67	11.65	10.15	8.94
10	9.90	12.09	14.11	14.29	13.05	13.05	11.04	9.87
11	9.29	11.00	12.81	12.84	11.73	11.70	11.76	8.21
12	11.10	12.76	14.74	14.79	12.77	12.73	15.28	11.02
13	12.96	14.70	15.64	15.76	14.43	14.43	14.28	13.06
14	8.96	10.81	12.24	12.34	11.34	11.31	9.85	9.81
15	10.40	12.08	13.36	13.52	12.66	12.68	11.43	10.96
16	13.10	15.72	17.43	17.58	16.45	16.45	12.72	14.17
17	9.19	10.88	11.94	12.01	10.65	10.63	12.94	10.72
18	7.23	8.74	9.78	9.84	8.80	8.81	9.56	8.30
19	9.95	12.08	13.04	13.19	11.51	11.53	13.29	11.15
20	8.11	9.66	11.44	11.54	9.69	9.69	8.43	9.91
21	9.86	11.69	12.67	12.68	11.36	11.36	13.65	11.23
22	11.34	13.04	13.89	13.96	12.34	12.32	16.26	12.59
23	8.29	9.58	10.42	10.58	9.16	9.22	14.82	8.78
24	10.13	11.70	12.87	12.89	11.20	11.19	15.51	11.28
25	12.33	13.44	13.87	13.88	12.24	12.45	19.26	12.93
26	12.48	14.68	15.46	15.56	12.97	12.98	16.99	14.06
27	11.11	12.65	13.64	13.59	12.01	12.00	18.84	12.35
28	10.02	11.60	12.39	12.44	10.91	10.97	14.89	11.07
29	11.28	12.96	14.24	14.40	12.45	12.43	17.81	12.05
30	11.43	13.11	14.24	14.48	11.08	11.08	23.12	12.27
31	14.51	17.03	18.55	18.82	16.25	16.25	22.79	16.81

A = 50snp_500 kb _100snp_0_0; B = 50snp_500 kb _100snp_0_2; C = 50snp_500 kb _100snp_1_2; D = 50snp_500 kb _100snp_1_5; E = 50snp_500 kb _15snp_0_1; F = 50snp_500 kb _50snp_0_1; G = 500snp_500 kb _50snp_0_1; H = pruned_50snp_500 kb _50snp_0_1 (FORMAT: “sliding window size”_”minimum length (kb) for a run to be called as a ROH”_”minimum number of SNPs for a run to be called as a ROH”_”number of heterozygous SNPs allowed in a ROH”_”number of missing calls allowed in a ROH”)

**Table 4:** Previously reported QTL for reproduction and health trait in the horse that overlap with common (> 95% of the sample) ROH regions in the Norwegian–Swedish Coldblooded Trotter

QTL ID	Symbol	Trait name	Chr	Start position (bp)	End position (bp)
103450	MOTSCT	Number of motile sperm	1	53958169	53958209
32144	OSTEO	Osteochondrosis	2	11816213	21391792
32142	OSTEO	Osteochondrosis	2	12910010	21391792
32146	OSTEO	Osteochondrosis	2	12910010	21391792
32145	OSTD	Osteochondrosis dissecans	2	13028376	22500086
32143	OSTEO	Osteochondrosis	2	18664801	19717761
32147	OSTEO	Osteochondrosis	2	18664801	23235964
29325	OSTEO	Osteochondrosis	3	105163057	105163097
29326	OSTEO	Osteochondrosis	3	105546982	105547022
29327	OSTEO	Osteochondrosis	3	105830605	105830645
29306	SUSBITE	Insect bite hypersensitivity	4	43000811	43945687
37902	OSTD	Osteochondrosis dissecans	5	77424966	77425006
29287	SUSBITE	Insect bite hypersensitivity	11	22016942	22985500
29268	RHOD	Rhodococcus equi infection	14	3055253	3055293
29315	SUSBITE	Insect bite hypersensitivity	15	20012397	20994475
29316	SUSBITE	Insect bite hypersensitivity	15	32000266	32987009
29035	GPT	Guttural pouch tympany	15	53093059	53093099
29111	GPT	Guttural pouch tympany	15	65298904	65298944
29067	GPT	Guttural pouch tympany	15	78013499	78013539
28922	OSTEO	Osteochondrosis	16	1299549	5389006
29002	OSTD	Osteochondrosis dissecans	16	1299549	5389006
28933	OSTEO	Osteochondrosis	16	5228939	5496903
28999	OSTD	Osteochondrosis dissecans	16	5228939	5496903
28927	OSTD	Osteochondrosis dissecans	16	22275834	22702331
28937	OSTEO	Osteochondrosis	16	22275834	22702331
29006	OSTEO	Osteochondrosis	16	22275834	22702331
29338	RER	Recurrent exertional rhabdomyolysis	16	29314251	29314291
29277	RER	Recurrent exertional rhabdomyolysis	16	29349222	29349262
29337	RER	Recurrent exertional rhabdomyolysis	16	29349222	29349262
29320	SUSBITE	Insect bite hypersensitivity	19	21037979	21977304
29298	SUSBITE	Insect bite hypersensitivity	20	41031989	41982509
37895	SUSBITE	Insect bite hypersensitivity	20	41530793	42603867
37896	SUSBITE	Insect bite hypersensitivity	20	41530793	42603867
28920	SARRESI	Equine sarcoids	23	16126529	41049320
28921	SARRESI	Equine sarcoids	25	24227654	30109054

## Discussion

As expected based on previous studies in other species, the realized F_ROH_ in the NSCT population tended to be slightly higher than the F_PED_ estimates [1–7, 17, 26]. However, in our study, applying strict threshold settings regarding the number of heterozygous SNPs or missing calls allowed in a ROH significantly reduced correlations between F_PED_ and F_ROH_, and drastically altered the ability to capture longer ROH. Since size and frequency of ROH provide evidence for relatedness within and between populations, as well as details on distant and recent ancestry, the ability to capture consistently long ROH is essential for the integration of genomic data into breeding evaluation and preservation protocols for the NSCT breed [4–6, 18–20]. Shorter ROH (< 1 Mb) tended to be more easily detected regardless of the ROH criteria applied, but longer ROH (> 10 Mb) were more difficult to capture when no heterozygous SNPs or missing calls were allowed in a ROH and at least 100 SNPs were required for a run to be called as a ROH. Although this seems logical since a true ROH does not include any heterozygous SNPs, the high-density equine genotyping array contains more than 670,000 SNPs. Even a genotyping error rate of only 1% could yield 6700 possibly incorrectly genotyped SNPs. Since these incorrectly genotyped SNPs, which are likely attributable to poor sample quality in the current study, tend to be randomly scattered across the entire genome, individual horses can be disproportionately affected simply by chance.

Nevertheless, regardless of the ROH threshold settings applied, F_ROH_ in the NSCT breed appears to have steadily increased between 2000 and 2009. While the overall inbreeding level within the breed is slightly underestimated based on classical metrics, the upward trend of inbreeding level revealed by the F_PED_ calculations is clearly supported by the F_ROH_ estimates and likely warrants additional exploration by the NSCT breeding industry—particularly in relation to the difference in inbreeding levels between Norwegian born horses and Swedish born horses (Table 2). Furthermore, it is important to note the difference in F_PED_ between the entire population and the sample of individuals used in our study (Fig. 1). Generally speaking, inbreeding is expected to increase by 1% per generation (i.e. 7–9 years in the NSCT). This 1% increase in inbreeding level is clearly seen in the F_PED_ values for the whole population, but is not so obvious for the sample of individuals analyzed here, for which a ~ 1.5% increase was observed instead of the expected 1% over the same time period. Consequently, since the sample of individuals used in our study included only raced horses, although, not certain, it is plausible that the population of raced NSCT is perhaps slightly more inbred than the unraced population.

Although NSCT is not currently considered an at risk breed, it represents unique Norwegian and Swedish genetic resources and is present on the department of agriculture’s list of horse breeds that should be preserved [27]. The NSCT industry has historically been at the forefront regarding the application of emerging genetic technologies in racehorses, and is currently providing F_PED_ estimates, as well as estimated breeding values (EBV) for breeders and owners to use as part of their criteria for determining sire/dam pairing [28, 29]. While this information has undoubtedly proved valuable over the last half-century, genomic information provides the opportunity to manage NSCT breeding more effectively - particularly if it is used to produce genomic EBV. In addition, the use of genomic information to determine both inbreeding levels and relationships between individuals is also likely to have a knock-on effect on performance, increasing the accuracy of the industry’s current EBV and therefore increasing the industry’s ability to improve the performance and health of their horses.

As with other species and breeds, opportunities exist for the NSCT industry to develop software programs that provide breeders with easily interpretable feedback on regions of the genome that are suboptimal from the perspective of genetic merit or that are sensitive to inbreeding within the population. Overall, 1403 common ROH regions were identified within the sample of raced horses used here. There were few overlaps with known QTL for health and reproduction traits, which indicates that perhaps only a small percentage of these regions may warrant concern, at this time [24]. Whereas multiple ROH regions (n = 17) contained QTL that are associated with osteochondrosis (OC) [30–33], it is possible that homozygosity in these regions may be optimal rather than detrimental when one considers the widely heralded robustness of the breed and that only raced horses were evaluated in our study. It is likely that both the draught horse origins of NSCT and the breeding industry’s emphasis on continued production of robust, tractable horses through artificial selection, have resulted in the breed displaying a strong resistance to the development of OC with increasing homozygosity in specific areas of the genome over time. A similar observation can also be made for the common ROH that overlap with QTL associated with recurrent exertional rhabdomyolysis (RER), which is another condition rarely seen in NSCT [34]. However, additional research is required to confirm this.

Increased inbreeding within a population also tends to impact fertility traits unfavorably; however, only one of the common ROH regions overlapped with a known QTL related to reproduction [35], which suggests that, at present, poor fertility may not be a major concern in the NSCT breed. Nevertheless, it is strongly recommended that future genomic studies in this breed should consider the inclusion of data on fertility traits, since it will likely prove to be highly beneficial in subsequent efforts to preserve the breed’s genetic variability in the long term [5, 36].

## Conclusions

In the current study, both F_PED_ and F_ROH_ were calculated for a sample of raced NSCT with F_ROH_ resulting in higher inbreeding coefficients, and both methods showing a gradual increase in inbreeding between 2000 and 2009. Stricter ROH threshold criteria regarding the number of heterozygous SNPs and missing calls allowed in a ROH significantly reduced correlations between F_PED_ and F_ROH_ and noticeably altered the chances of capturing long ROH. While the exact reasons behind this decrease in correlations are not known with certainty, the established associations between classical F estimates and recent inbreeding within a pedigree (characterized by long ROH) in other species provide some insight. Since retaining genetic variation is important to allow populations to adapt to changing environments, the integration of genomic data into their EBV and the use of molecular data to identify both genomic regions contributing to inbreeding depression and pedigree errors will likely prove invaluable as the NSCT industry moves forward in its conservation and selection efforts.

## Additional files

**Additional file 1: Table S1.** Threshold settings used to define runs of homozygosity in PLINK and the corresponding average F_ROH_ for a sample of raced Norwegian-Swedish Coldblooded trotters born between 1 January 2000 and 31 December 2009.

**Additional file 2: Table S2.** Correlation matrix between F_PED_ and all F_ROH_ estimates.

**Additional file 3: Table S3.** Results of the paired t-test (P-values) between F_PED_ and all F_ROH_ estimates.

**Additional file 4: Figure S1.** Histograms for run of homozygosity (ROH) lengths based on four different threshold combinations in PLINK v 1.07. A = 50snp_500kb _100snp_0_0; B = 50snp_500kb _100snp_0_2; C = 50snp_500kb _100snp_1_2; D = 50snp_500kb _100snp_1_5 (FORMAT: “sliding window size”_”minimum length (kb) to be called as homozygous”_”minimum number of SNPs to be called as homozygous”_”number of heterozygotes allowed”_”number of missing calls allowed”).

**Additional file 5: Figure S2.** Histograms for run of homozygosity (ROH) lengths based on varying window size thresholds in PLINK v 1.07. A = 50snp_500kb _15snp_0_1; B = 50snp_500kb _50snp_0_1; C = 500snp_500kb _50snp_0_1; D = pruned_50snp_500kb _50snp_0_1 (FORMAT: “sliding window size”_”minimum length (kb) for a run to be called as a ROH”_”minimum number of SNPs for a run to be called as a ROH”_”number of heterozygous SNPs allowed in a ROH”_”number of missing calls allowed in a ROH”).

**Additional file 6: Table S4.** Homozygous regions of the genome that are shared by at least 95% of the sample of Norwegian-Swedish Coldblooded Trotters (n = 566).

## Data Availability

The data that support the findings of this study are available from the Swedish Trotter Association (Stockholm, Sweden) and the Norwegian Trotter Association (Oslo, Norway), but restrictions apply to the availability of these data, which were used under license for the current study, and so are not publicly available. However, data are available from the authors upon reasonable request and with permission of the Swedish Trotter Association (Stockholm, Sweden) and the Norwegian Trotter Association (Oslo, Norway).
