# Supplementary figures and images for: Genomic measures of inbreeding in the Norwegian–Swedish Coldblooded Trotter and their associations with known QTL for reproduction and health traits
Source: Genet Sel Evol. 2019 May 27;51:22. doi: 10.1186/s12711-019-0465-7 (PMC6537210; doi:10.1186/s12711-019-0465-7)

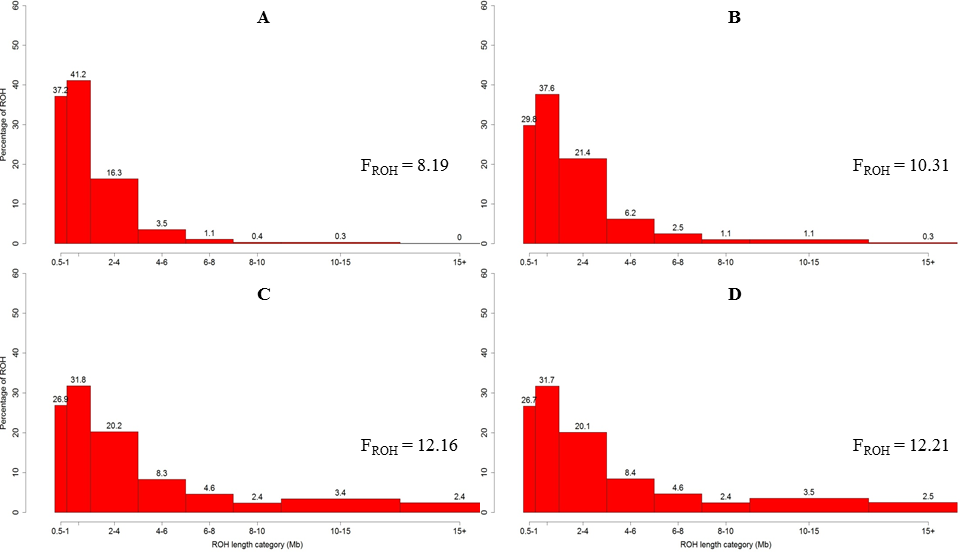

Supplement: Supplementary file 4 — Additional file 4: Figure S1. Histograms for run of homozygosity (ROH) lengths based on four different threshold combinations in PLINK v 1.07. A = 50snp_500kb _100snp_0_0; B = 50snp_500kb _100snp_0_2; C = 50snp_500kb _100snp_1_2; D = 50snp_500kb _100snp_1_5 (FORMAT: “sliding window size”_”minimum length (kb) to be called as homozygous”_”minimum number of SNPs to be called as homozygous”_”number of heterozygotes allowed”_”number of missing calls allowed”). [file 12711_2019_465_MOESM4_ESM.tif]

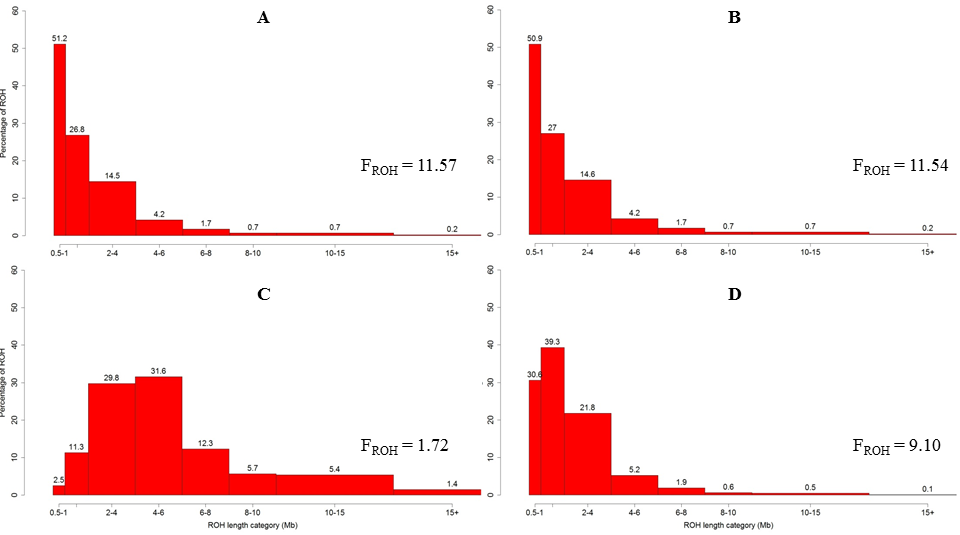

Supplement: Supplementary file 5 — Additional file 5: Figure S2. Histograms for run of homozygosity (ROH) lengths based on varying window size thresholds in PLINK v 1.07. A = 50snp_500kb _15snp_0_1; B = 50snp_500kb _50snp_0_1; C = 500snp_500kb _50snp_0_1; D = pruned_50snp_500kb _50snp_0_1 (FORMAT: “sliding window size”_”minimum length (kb) for a run to be called as a ROH”_”minimum number of SNPs for a run to be called as a ROH”_”number of heterozygous SNPs allowed in a ROH”_”number of missing calls allowed in a ROH”). [file 12711_2019_465_MOESM5_ESM.tif]
